# Identification and Characterization of Wheat Yellow Striate Virus, a Novel Leafhopper-Transmitted Nucleorhabdovirus Infecting Wheat

**DOI:** 10.3389/fmicb.2018.00468

**Published:** 2018-03-14

**Authors:** Yan Liu, Zhenzhen Du, Hui Wang, Song Zhang, Mengji Cao, Xifeng Wang

**Affiliations:** ^1^State Key Laboratory for Biology of Plant Diseases and Insect Pests, Institute of Plant Protection, Chinese Academy of Agricultural Sciences, Beijing, China; ^2^National Citrus Engineering Research Center, Citrus Research Institute, Southwest University, Chongqing, China

**Keywords:** wheat yellow striate virus, transcriptome sequencing, leafhopper, *Nucleorhabdovirus*, transmission electron microscopy

## Abstract

A new wheat viral disease was found in China. Bullet-shaped viral particles within the nucleus of the infected wheat leave cells, which possessed 180–210 nm length and 35–40 nm width, were observed under transmission electron microscopy. A putative wheat-infecting rhabdovirus vectored by the leafhopper *Psammotettix alienus* was identified and tentatively named wheat yellow striate virus (WYSV). The full-length nucleotide sequence of WYSV was determined using transcriptome sequencing and RACE analysis of both wheat samples and leafhoppers *P. alienus*. The negative-sense RNA genome of WYSV contains 14,486 nucleotides (nt) and seven open reading frames (ORFs) encode deduced proteins in the order N-P-P3-M-P6-G-L on the antisense strand. In addition, WYSV genome has a 76-nt 3′ leader RNA and a 258-nt 5′ trailer, and the ORFs are separated by conserved intergenic sequences. The entire genome sequence shares 58.1 and 57.7% nucleotide sequence identity with two strains of rice yellow stunt virus (RYSV-A and RYSV-B) genomes, respectively. The highest amino acid sequence identity was 63.8% between the L proteins of the WYSV and RYSV-B, but the lowest was 29.5% between the P6 proteins of these viruses. Phylogenetic analysis firmly established WYSV as a new member of the genus *Nucleorhabdovirus*. Collectively, this study provided evidence that WYSV is likely the first nucleorhabdovirus described infecting wheat via leafhopper *P. alienus* transmission.

## Introduction

More than 50 viruses are known to infect wheat (*Triticum aestivum* L.) worldwide, causing severe symptoms that can decrease yield and quality (Ordon et al., 2009). Most wheat-infecting viruses are transmitted by an insect vector, including aphids, planthoppers and leafhoppers (Nault and Ammar el, 1989; Hogenhout et al., 2008; Whitfield et al., 2015; Zhang et al., 2017a). The most important vector-transmitted viruses that infect wheat are the aphid-transmitted barley yellow dwarf virus (BYDV, genus *Luteovirus*) and cereal yellow dwarf virus (CYDV, genus *Polerovirus*) (Miller and Rasochova, 1997; Liu et al., 2007), and the leafhopper-transmitted wheat dwarf virus (WDV, genus *Mastrevirus*) (Manurung et al., 2004; Schubert et al., 2007; Wang et al., 2014). In addition, four cytorhabdoviruses have been described infecting wheat: northern cereal mosaic virus (NCMV) (Tanno et al., 2000) and barley yellow striate mosaic virus (BYSMV) (Yan et al., 2015), which are transmitted by the small brown planthopper (Di et al., 2014). A new cytorhabdovirus infecting wheat, named as maize yellow striate virus, was recently described (Maurino et al., 2018). Furthermore, other cytorhabdovirus, named as wheat American striate mosaic virus (WASMV) has been described infecting wheat transmitted by leafhopper (Jackson et al., 2005).

Plant-infecting rhabdoviruses are included in four genera: *Cytorhabdovirus, Nucleorhabdovirus, Dichorhavirus*, and *Varicosavirus* (Amarasinghe et al., 2017; Dietzgen et al., 2017). Dichoravirus and Varicosavirus members have a bipartite genome whereas Nucleo and Cytorhabdovirus members are monopartite. Cytorhabdovirus or Nucleorhabdovirus were classified based on whether viral replication occur in the cytoplasm or the nuclei of infected plant and insect vector cells (Jackson et al., 2005; Hogenhout et al., 2008; Ammar el et al., 2009). Nucleorhabdoviruses are mainly transmitted by leaf- or planthoppers whereas cytorhabdoviruses are transmitted by aphids or planthoppers (Mann and Dietzgen, 2014). To date, over 20 complete genomic sequences are available for plant-infecting rhabdoviruses (Dietzgen et al., 2017). Generally, the genomes of monopartite plant rhabdoviruses are 11–15 kb in size and have the same organization as their animal-infecting counterparts, which encode at least five proteins: nucleocapsid protein (N), phosphoprotein (P), matrix protein (M), glycoprotein (G) and RNA-dependent RNA polymerase (L) in the order 3′-N-P-M-G-L-5′ with some additional accessory genes, which are usually interspersed between N-P, P-M and G-L genes (Kuzmin et al., 2009; Walker et al., 2011, 2015).

A new wheat disease was observed in in April 2016 in Hancheng, Shaanxi Province, China. Infected wheat plants showed yellowing and mild chlorotic streaks along small veins on leaves. After field-collected leafhoppers (*Psammotettix alienus*; Hemiptera: Cicadellidae), common pests of wheat fields, were allowed to feed on healthy wheat seedlings in a greenhouse, similar disease symptoms developed after 2–3 weeks, suggesting the causal agent of this new disease is a virus that is transmitted by the leafhopper *P. alienus*.

Here we present the discovery of a novel virus in wheat plants, transmitted by leafhopper, using the RNA sequencing (RNA-Seq) platform and electron microscopy. Moreover, we analyzed the genome organization and phylogeny of this virus. We also tested the effect of the virus on the host plants and the specificity of the vector. Our results reveal that the wheat-infecting novel virus is a single-stranded (ss) negative- sense RNA virus, provisionally named as wheat yellow striate virus (WYSV), which should be classified as a new member of the genus *Nucleorhabdovirus* of the family *Rhabdoviridae*.

## Materials and methods

### Virus source and maintenance

In April 2016, viruliferous leafhoppers *P. alienus* were originally collected from wheat fields in Hancheng (Shaanxi Province, China) and subsequently maintained in insect-proof cages. Wheat seedlings (cv. Yangmai 12) at the single-leaf stage in the greenhouse were used for inoculations and then grown in the growth chambers at 22 ± 1°C with 16 h daylight. Symptomatic plants after 3 weeks were used as inoculum to propagate the virus. Non-viruliferous leafhoppers collected from a healthy wheat field in 2014 were separately reared on 3-leaf-stage seedlings. Cohorts of about 60 adult leafhoppers were allowed to feed on symptomatic plants for a 72 h acquisition access period (AAP), then released on healthy wheat seedlings for 98 h, and finally leafhoppers were transferred to new wheat seedlings (three insects per plant). After a 72 h inoculation access period (IAP), leafhoppers were killed by applying 1:1000 diluted 10% imidacloprid wettable powder. Corresponding treatment that non-viruliferous leafhoppers exposed to healthy plants served as the control.

### Host range and insect vector transmission assays

Transmission of WYSV was tested for several insects, including three species of planthoppers, *Nilaparvata lugens, Laodelphax striatellus* and *Sogatella furcifera* Horváth and three aphid species including *Sitobion avenae, Schizaphis gramienum*, and *Rhopalosiphum padi*. Only one leafhopper *P. alienus* tested because it is widely distributed in the Northwest and North of China, which are main areas of wheat production and was the only leafhopper identified in WYSV-infected fields. Barley cv. Longpi 3 and oat cv. Coast Black were chosen to preliminarily determine the host range. Rearing, acquisition and transmission experiments were carried out as described above. All test plants were maintained in an illuminated insect-containment incubator for 2–3 weeks, then plants were observed for disease symptoms.

### Electron microscopy

Leaf pieces from WYSV-infected wheat plants were fixed with glutaraldehyde, postfixed with 1% osmium tetroxide and embedded in araldite CY212 (Agar Scientific, Standsted, UK). Ultrathin sections were double-stained in 5% w/v uranyl acetate and 2% w/v lead citrate (pH 12) before observation and examined in a Hitachi model H-7500 transmission electron microscope (TEM, Hitachi High-Technologies, Tokyo, Japan) and photographed.

### Next-generation sequencing and sequence assembly

For deep sequencing, total RNA was extracted from infected wheat leaves and from viruliferous leafhoppers using TRIzol reagent (Invitrogen, USA) according to the manufacturer's protocol. RNA quality and concentration were determined with an Agilent 2100 Bioanalyzer (plant RNA Nano Chip, Agilent, USA). cDNA libraries prepared from ribo-depleted RNA samples were subjected to NGS using the Illumina HiSeq X-ten and a paired-end 150 bp set-up. Both libraries were constructed and sequenced at Berry Genomics Bioinformatics Technology Co., Beijing, China. To analyze the RNA-seq of infected wheat, the sequence reads from the cDNA library were initially mapped to the reference genome of *T. aestivum* cv. Chinese Spring (http://www.wheatgenome.info/wheat_genome_databases.php) (Montenegro et al., 2017). Reads not mapped to the reference genome were used for further analysis. To analyze the transcriptome of the leafhopper *P. alienus*, the Illumina reads were *de novo* assembled using the Trinity program (Haas et al., 2013) after raw reads from the platform were trimmed of adaptor sequences and low-quality reads by the CLC Genomics Workbench 9.5 (Qiagen, Valencia, CA, USA). The assembled contiguous sequences (contigs) were used as queries using the BLAST suite of programs from the National Center for Biotechnology Information (NCBI) with standard parameters (https://blast.ncbi.nlm.nih.gov/Blast.cgi).

### RACE analysis and validation of the virus genomes

To determine the terminal sequence of the WYSV genomic RNA, the 3′ and 5′ ends were amplified using the 3′ and 5′ RACE System for Rapid Amplification of cDNA Ends (Life Technologies). RACE-PCR bands were purified using the Wizard SV Gel and PCR Clean-Up System (Promega, Madison, WI, USA) and cloned into the pEASY-T5 vector (TransGen Biotech, Beijing, China). At least six clones from each subclone were sequenced (Taihe Biotechnology Co., Beijing, China). The complete genomic sequence was confirmed by re-sequencing several fragments that covered the whole genome, which were amplified by RT-PCR using specific primers (Table S1).

### Bioinformatics analysis

Sequences were assembled and analyzed using Vector NTI 11.5 software, then directly submitted to the GenBank database of the NCBI. ORFs in the reconstructed genome were predicted using the ORF Finder program of the NCBI. Conserved and functional domains of the predicted proteins in WYSV were identified using the Conserved Domain Database (CDD) of the NCBI (Marchler-Bauer et al., 2015) and the SMART tool (Letunic and Bork, 2018). A functional motif search was carried out against the pfam database (Finn et al., 2016) and PROSITE database at http://prosite.expasy.org/. Identity analyses were performed using the needle program in EMBL-EBI (https://www.ebi.ac.uk/Tools/psa/emboss_needle/); transmembrane helices were predicted using the online server TMHMM Server v. 2.0 (http://www.cbs.dtu.dk/services/TMHMM/), and SignalP were used to predict signal peptide cleavage sites (http://www.cbs.dtu.dk/services/SignalP/). The nuclear localization and export signals were predicted by cNLS Mapper (Kosugi et al., 2009) and NetNES 1.1 (La Cour et al., 2004), respectively. Mapping of clean RNA-seq reads from both wheat and leafhopper to the complete viral genome sequence as a reference was performed using the CLC Genomics Workbench 9.5. Evolutionary relationships of WYSV with representative rhabdoviruses were inferred by construction of phylogenetic trees using the MEGA 7.0 program with 1000 bootstrap replications (Kumar et al., 2016).

## Results

### Biological properties of WYSV

**Figure 1:**
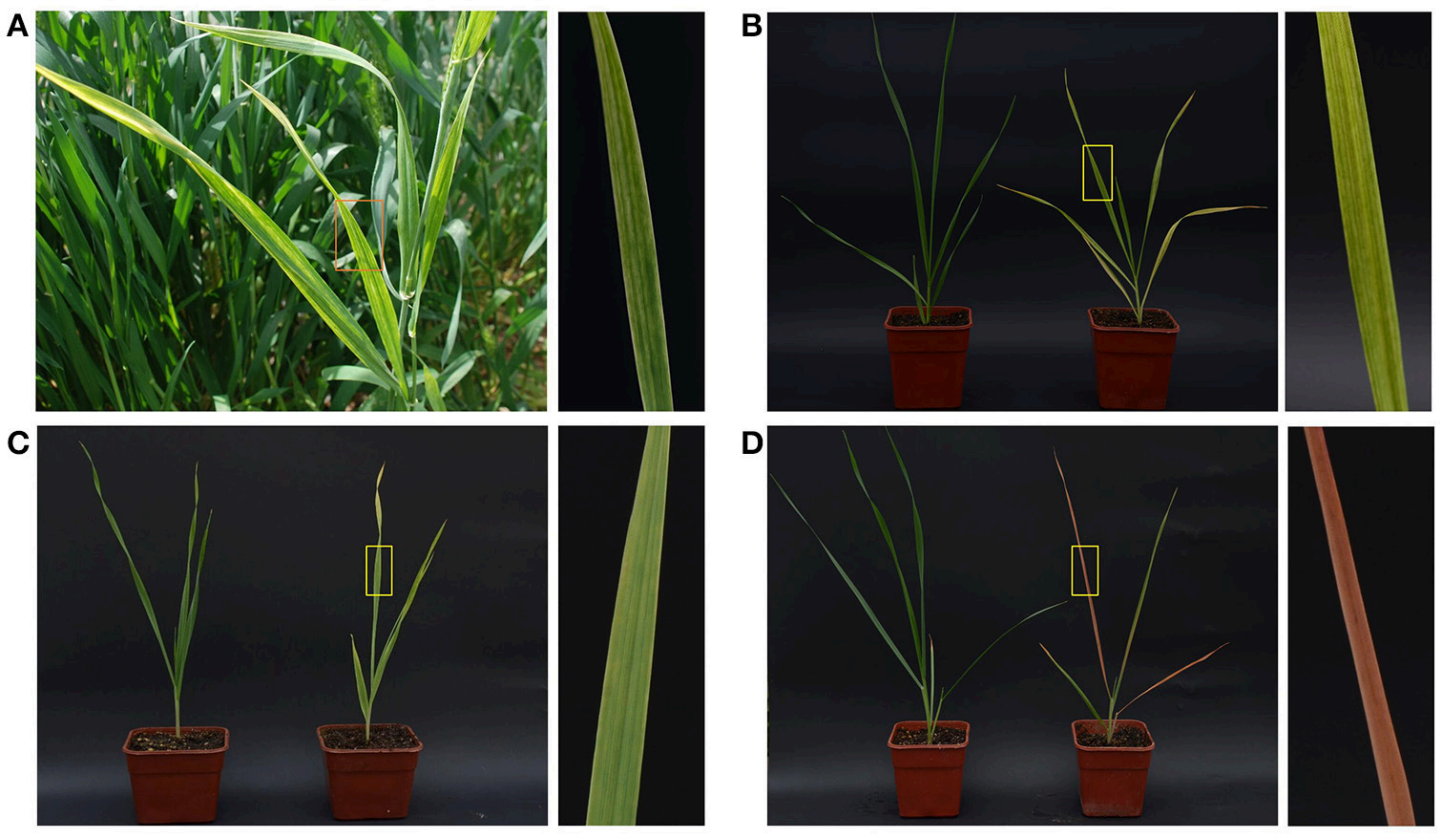
Symptoms of WYSV-infected plant in the field **(A)** and WYSV-infected wheat **(B)**, barley **(C)**, and oat **(D)** after viruliferous leafhoppers were allowed to feed on the plant. The healthy controls plants are shown on the left of panels **B–D**, respectively. Close up of leaf symptoms are shown to the right of each panel.

During a survey of virus diseases in April 2016, wheat plants with unusual symptoms—yellow stripes on leaves and slight dwarfism—were observed at Hancheng, Shaanxi Province, which is the most important wheat-growing area of Northwest China (Figure 1A). The collected samples were tested by PCR using WDV specific primers, and then negative samples were used for further study. About 2000 individuals of *P. alienus*, the main pest in the local fields, were captured to rear on healthy wheat plants cv. Yangmai 12 in an insect-free greenhouse. After 3 weeks, over 80% plants showed similar symptoms to those observed on the infected plants in the field (Figure 1B). WYSV was also effectively transmitted by *P. alienus* to other cereal crops including barley and oat; after 3 weeks, leaves of barley cv. Longpi 3 had become golden yellow (Figure 1C), and those of oat cv. Coast Black were deep-red (Figure 1D). In addition, WYSV was transmitted to wheat plants only by *P. alienus*, not by the brown planthopper (*N. lugens*), small brown planthopper (*L. striatellus*), white-backed planthopper (*S. furcifera*) and the three species of wheat aphids (*S. avenae, S. gramienum* and *R. padi*), which indicated strong virus-vector specificity.

### Virion morphology and cytopathology

**Figure 2:**
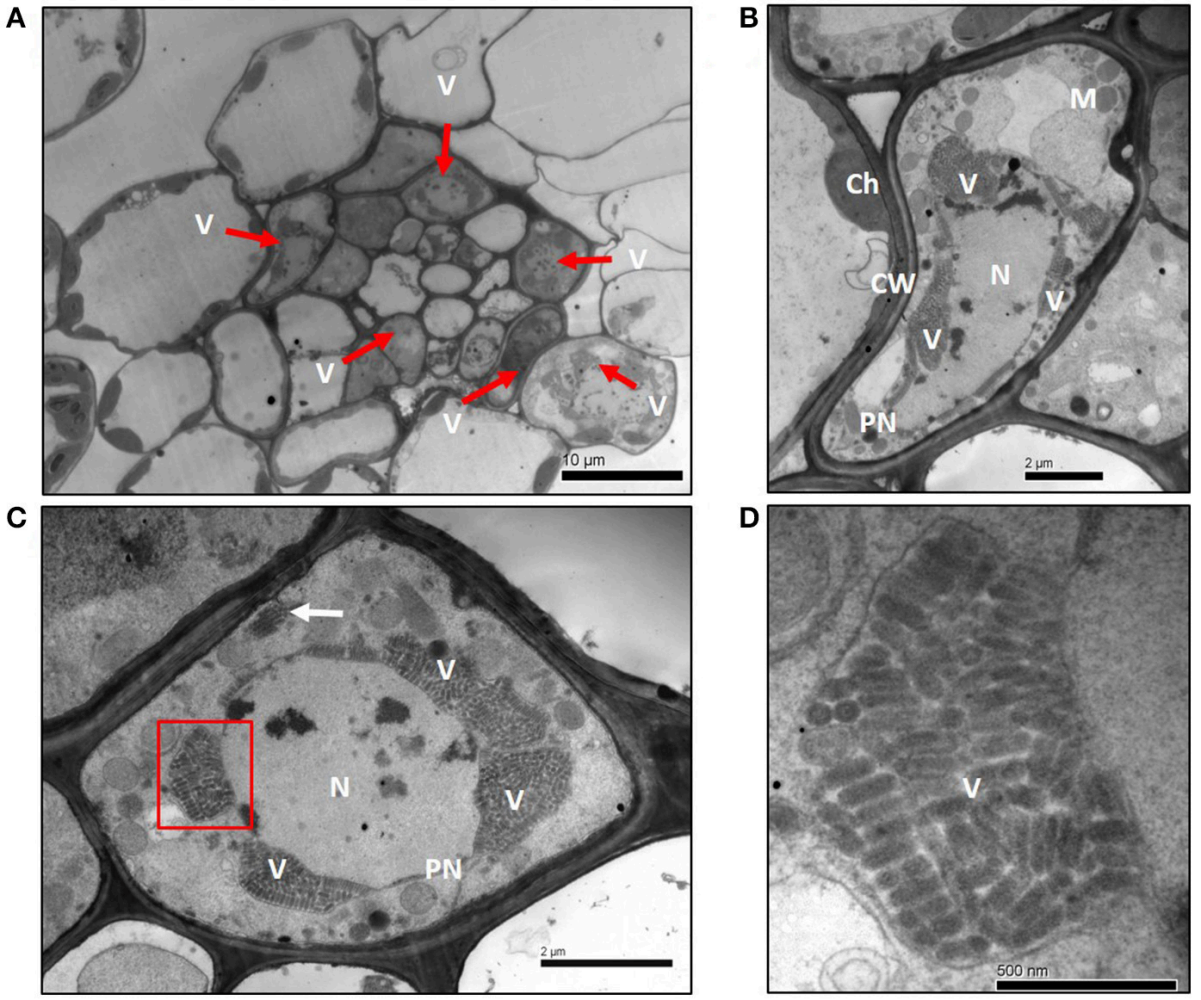
Electron micrographs of thin sections of wheat leaf tissue infected with WYSV at 15 dpi. **(A)** Infected cells were located mainly in the vascular bundle sheath cells of leaves. **(B,C)** Cluster of virus particles accumulated at the perinuclear regions. A few viral particles aggregated in vesicles (white arrow) near the cell wall (panel **C**). **(D)** Magnified virus particles from section (indicate with a red box) of panel **C**. Ch, chloroplast; CW, cell wall; N, nucleus; PN, perinuclear regions; M, mitochondria; V, virions.

When ultrathin sections of infected wheat plant leaf tissue were observed under the TEM, typical bullet-shaped virions of 180–210 nm length and 35–40 nm width were found in the nucleus (Figures 2A–D). The morphology and structure of these virions were closely similar to other plant rhabdoviruses (Franco et al., 1980; Jackson et al., 2005). Vascular tissue of infected wheat plant contained many infected cells, and the virus particles were predominantly in the nucleus of infected cells (Figure 2A). Clustered viral particles were observed to occur within the expanded perinuclear space (Figures 2B,C), which is similar to the localization of other nucleorhabdoviruses (Redinbaugh et al., 2002; Ammar et al., 2005). The TEM results support the classification of WYSV as a tentative nucleorhabdovirus.

### Sequence assembly

Two rRNA-depleted RNA-seq libraries, generated from symptomatic leaves and viruliferous leafhoppers and sequenced by an Illumina Genome analyzer, resulted in 103,396,270 and 61,864,662 raw paired-end 150-bp reads, respectively. All raw reads were filtered according to quality, and 57,220,212 (55.34%) of these reads from the wheat samples were screened out after mapping to the wheat genome. *De novo* assembly yielded 259,179 contigs of 200–19,963 nt and 466,904 contigs of 200–28,405 nt, respectively. All contigs were analyzed by BlastN and BlastX and two contigs of 14,512 for wheat and 14,399 for leafhopper which share identity with other plant rhabdoviruses were selected to obtain the WYSV whole genome.

### WYSV genome analysis

**Figure 3:**
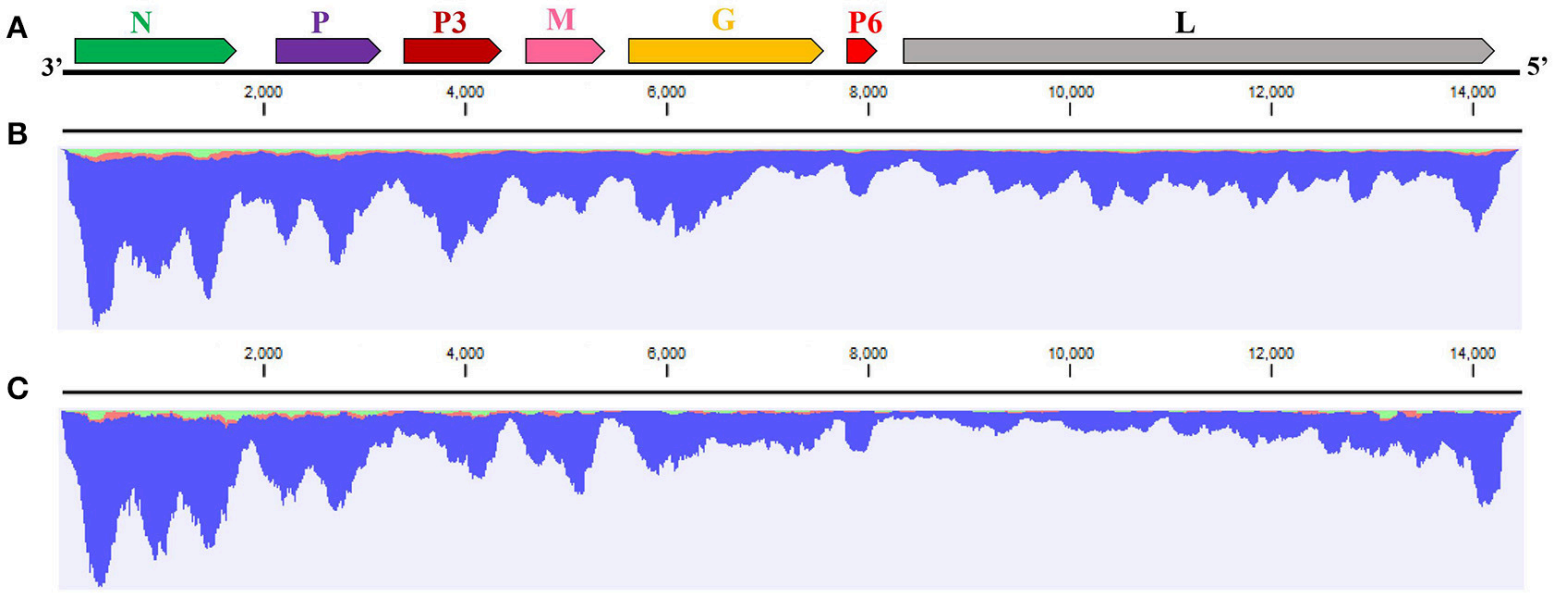
Analysis of WYSV genome. **(A)** Genome organization of WYSV. Each ORF (N, P, P3, M, G, P6, and L genes are represented by arrowed rectangles at the top; relative gene sizes are shown) was arranged in the 3′-5′ negative sense. RNA-seq mapping for **(B)** wheat and **(C)** the leafhopper showed fluctuating read distributions on viral genomic RNA.

**Figure 4:**
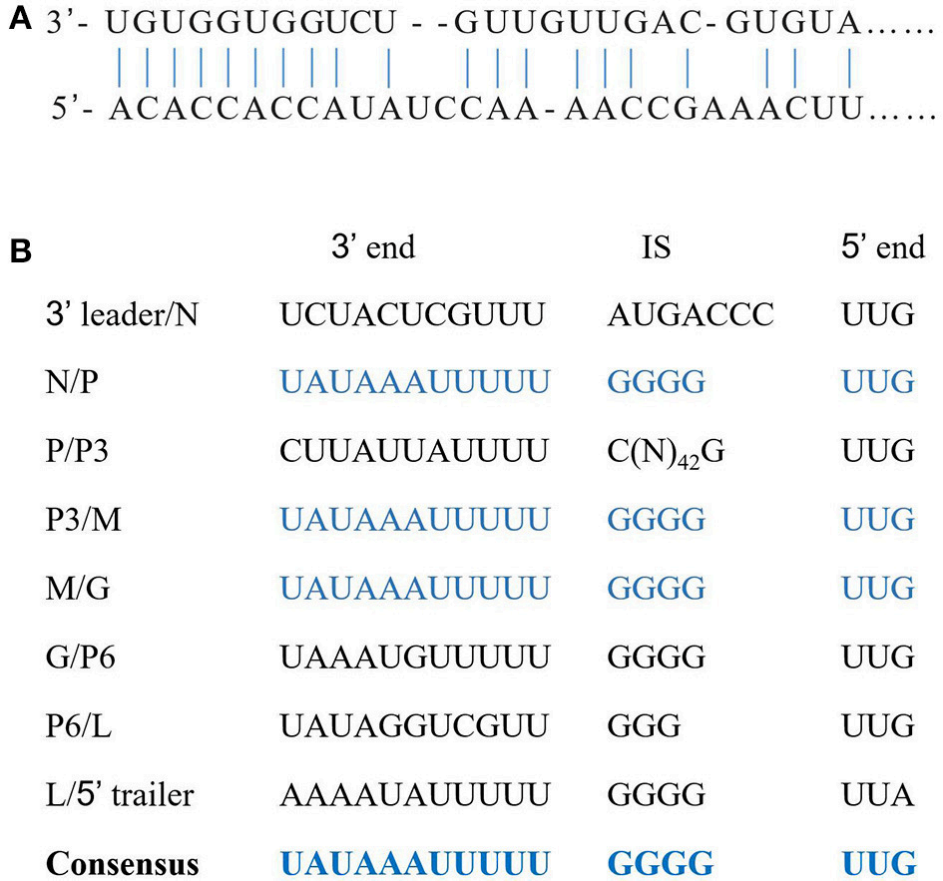
Sequence features of the WYSV genome. **(A)** Complementary structure between the 3′ and 5′ termini in the genome. Vertical lines indicate nucleotides that are complementary between the leader and trailer sequences. **(B)** Intergenic regions and conserved gene junctions in the viral sense orientation of WYSV. Nucleotides corresponding to the 3′ end of the mRNAs (3′ end), the intergenic sequences (IS), and the 5′ end of the following mRNA (5′ end) are indicated.

The complete sequence of the WYSV negative-sense RNA genome is 14,486 nt long and was deposited in Genbank as accession MG604920. Blast analysis revealed that WYSV has seven ORFs with an arrangement from the 3′- to 5′-end: N (ORF1), P (ORF2), P3 (ORF3), M (ORF4), G (ORF5), P6 (ORF6), and L (ORF7) (Figure 3A). RNA-seq reads of both wheat (Figure 3B) and leafhopper (Figure 3C) mapped to the WYSV genome exhibited similar fluctuating distribution on the viral genomic RNA, and the reads in each ORF region were much more numerous than in the nearby untranslated region. The WYSV coding sequences were flanked by a 76-nt 3′ leader and a 258-nt 5′ trailer. The nine terminal nt for the 3′ leader and the 5′ trailer were complementary, and the base pairing between positions 1–20 and 14,467–14,486 in the viral negative-sense RNA, which had four base mismatches, can potentially form a putative panhandle structure common to rhabdovirus genomes (Figure 4A).

The features of the proteins encoded by the WYSV genome are shown in Table 1. Amino acid sequence comparisons revealed that WYSV has the closest relationship to RYSV (Table 2). The ORF1 is 1,635 nt long and encode the putative nucleocapsid protein, which has 544 aa with a predicted molecular weight of 59.8 kDa and an isoelectric point (pI) of 8.81. Pairwise comparisons with other selected rhabdoviruses indicated that the identity shared between the N proteins of WYSV and other nucleorhabdoviruses ranged from 17.6 to 50.2%. The aa sequence of the WYSV N protein putatively contains one nuclear localization signal (NLS) at the amino termini (aa positions 11–21) and two nuclear export signals (NESs) at positions 311 and 316. The second gene contains a 1,050-nt ORF, which encodes 349 aa putative phosphoprotein with a predicted molecular weight of 37.4 kDa (pI = 9.28). The aa sequence identities between the P proteins of WYSV and other nucleorhabdoviruses ranged from 6.2 to 42.3%. The ORF3, comprised of 1,008 nt, which encoded 335 aa protein 3 (P3) gene with and an estimated molecular mass of 37.7 kDa (pI = 8.82). Sequence identities between the P3 proteins of WYSV and other nucleorhabdoviruses ranged from 7.0 to 52.2% (aa). The aa sequence of the WYSV P3 protein putatively contains three NESs at positions 69, 72, and 74.

**Table 1:** Features of the ORFs encoded by the WYSV genome.

**ORF**	**Protein**	**ORF genomic location**	**ORF length (nt) ^a^**	**Protein length (aa) ^a^**	**Protein mass (kDa)**	**Isoelectric point**	**Highest scoring virus protein/e-value (Blast X)**
1	N	77-1711	1635	544	59.8	8.81	RYSV/0.0
2	P	2084-3133	1050	349	37.4	9.28	RYSV/2e-86
3	P3	3348-4355	1008	335	37.7	8.82	RYSV/2e-140
4	M	4563-5375	813	270	30.2	7.71	RYSV/3e-114
5	G	5581-7566	1986	661	74.9	5.30	RYSV/0.0
6	P6	7759-8103	345	114	13.1	3.95	RYSV/1.6
7	L	8331-14228	5898	1965	224.4	6.22	RYSV/0.0

a *nt, nucleotide; aa, amino acid*.

**Table 2:** Amino acid sequence identities (%) of WYSV proteins compared with those of other plant rhabdoviruses.

**Genus**	**Virus name and GenBank accession No**.	**N**	**P**	**P3**	**M**	**G**	**P6**	**L**
Nucleorhabdovirus	rice transitory yellowing virus (RYSV-B) [AB516283]	50.2	42.3	52.2	53.8	52.0	29.5	63.8
	rice yellow stunt virus (RYSV-A) [AB011257]	48.0	42.0	52.2	57.4	53.6	29.5	62.1
	taro vein chlorosis virus (TaVCV) [AY674964]	20.5	12.2	14.0	20.9	27.0	/	30.2
	maize Iranian mosaic virus (MIMV) [DQ186554]	18.0	11.8	13.2	16.0	25.1	/	31.3
	potato yellow dwarf virus (PYDV) [GU734660]	25.0	14.0	16.3	19.1	22.5	/	33.5
	eggplant mottled dwarf virus (EMDV) [KJ082087]	23.8	17.2	16.7	19.7	20.8	/	33.6
	sonchus yellow net virus (SYNV) [L32603]	18.7	6.2	15.9	8.4	22.4	/	25.5
	datura yellow vein virus (DYVV) [KM823531]	22.1	7.7	17.1	7.8	21.0	/	26.7
	maize fine streak virus (MFSV) [AY618417]	17.6	16.1	7.0	7.0	20.7	/	26.9
	maize mosaic virus (MMV) [AY618418]	22.3	11.3	12.7	1.7	24.5	/	32.0
Cytorhabdovirus	lettuce yellow mottle virus (LYMoV) [EF687738]	15.1	10.4	9.5	10.2	16.0	/	22.2
	alfalfa dwarf virus (ADV) [KP205452]	14.7	14.1	9.5	2.0	15.5	0.0	22.9
	lettuce necrotic yellows virus (LNYV) [AJ867584]	17.2	8.2	9.0	15.6	14.9	/	22.7
	colocasia bobone disease-associated virus (CBDaV) [KT381973]	14.5	9.0	11.6	16.3	14.8	/	23.0
	northern cereal mosaic virus (NCMV) [AB030277]	14.1	9.0	12.8	18.4	13.5	/	24.8
	barley yellow striate mosaic virus (BYSMV) [KM213865]	14.6	8.1	11.2	15.3	14.9	7.8	23.5
	colocasia bobone disease-associated virus(CBDaV) [KT381973]	14.5	9.0	10.9	16.3	14.8	/	23.0
Dichorhabvirus	coffee ringspot virus (CoRSV) [KF812525, KF812526]	17.6	3.6	16.9	9.6	16.9	/	26.7
	orchid fleck virus (OFV) [AB244417, AB244418]	19.3	8.7	16.0	6.6	18.0	/	29.1
Varicosavirus	lettuce big-vein associated virus (LBVaV) [AB114138, AB075039]	14.5	14.3	13.8	12.0	8.9	/	22.8

The 813-nt ORF4 likely encodes the matrix protein which has a size of 270aa and an estimated molecular mass of 30.2 kDa (pI = 7.71). This protein shares an aa sequence identity ranging from 1.7 to 57.4% when compared with M protein encoded other nucleorhabdoviruses. It is predicted that the WYSV M protein contains ten NESs at positions 28, 107–114, and 116.

**Figure 5:**
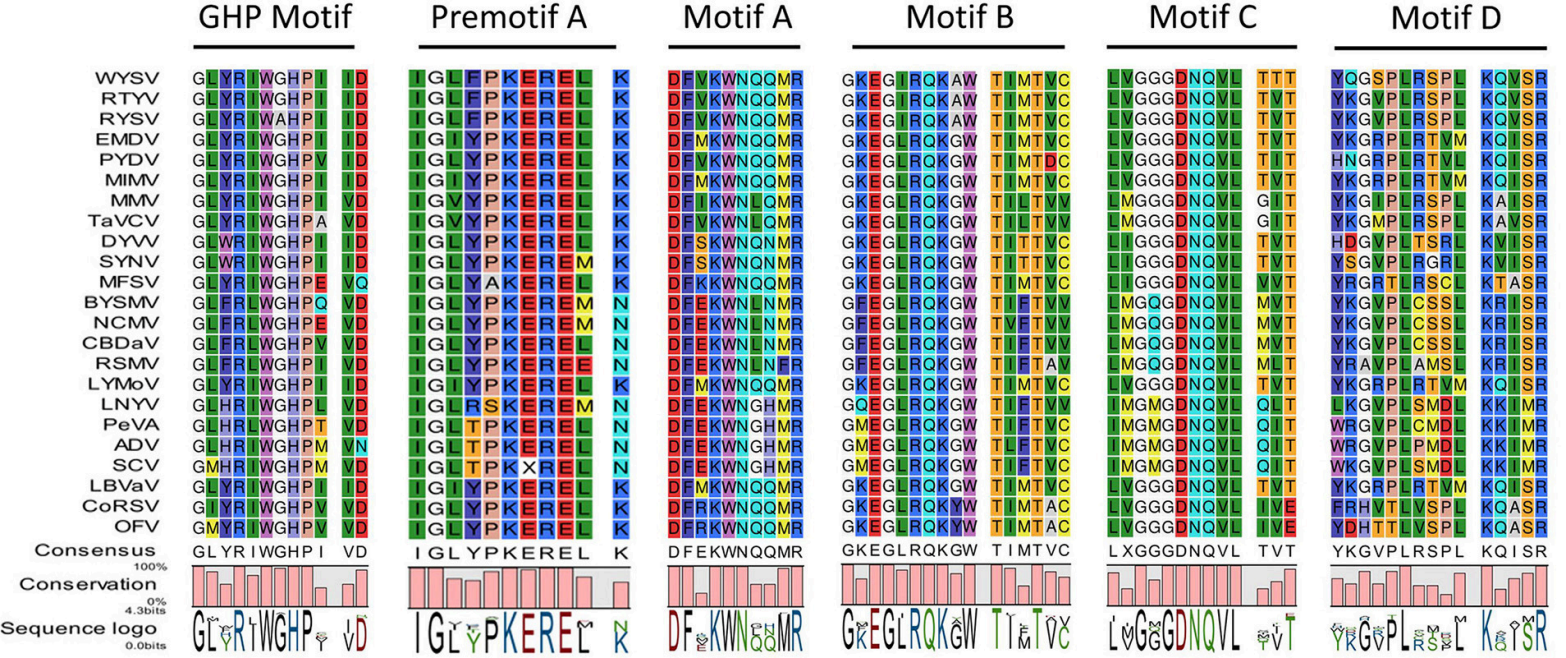
Amino acid alignment of conserved blocks within RdRp encoded by WYSV and selected plant rhabdoviruses. Conserved residues are highlighted in a different color.

The ORF5 which is 1,986 nt in length, encodes 661 aa putative glycoprotein with a predicted of 74.9 kDa (pI = 5.30), putatively contains two NESs at positions 10 and 17. It also contains two predicted transmembrane domains ^7^MLTIIICMLFGLYMIMLG^24^ and ^611^MLIIIVCLIGGYYVLIIPYGFLR^633^ at the N-terminal and C-terminal regions, respectively. The deduced G protein shares aa sequence identity with the other nucleorhabdovirus G proteins ranging from 20.7 to 53.6 %. In addition, the WYSV genome has an additional ORF with 114 aa encoding P6 on the vc strand, similar to RYSV. The P6 protein is 345 nt in length and has a calculated molecular mass of 13.1 KDa. The pairwise alignment of the deduced aa sequence of WYSV P6 protein revealed a similarity of only 29.5% to both RYSV-A and RYSV-B. This putative protein had no significant matches with ADV P6, RSMV P6 as well as BYSMV P9. According to the predicted pI (3.95), P6 is highly acidic, similar to P6 of RYSV-A (pI 3.49). It is predicted that the WYSV P6 protein contains one NES at position 78. ORF7 is 5,898 nt long and encodes a 1,965 aa putative L protein with a 224.4 KDa protein and a PI of 6.22. The putative L protein also contains two predicted transmembrane domains ^158^ADHPYVTCTISTVLFILSVLNNV^180^ and ^201^GNLVCLSLAAATLCYLSTDILVF^223^ at the N-terminus. The L protein shares 25.5 to 63.8% aa similarity with the L proteins of other of nucleorhabdoviruses. However, WYSV L protein is an acidic protein with a calculated pI of 6.22, which is similar that the pI described for RYSV, whereas the L proteins encoded by the other rhabdoviruses and most members of the other families of the Mononegavirales are alkaline. A NLS has been predicted at the amino termini of the WYSV L protein (aa positions 1823–1831). In addition, this putative L protein contains polymerase module motifs characteristic of the RNA-dependent RNA polymerases (RdRp) of negative-strand RNA viruses, consisting of six invariant regions (Figure 5) (Bourhy et al., 2005). The GHP (Gly-His-Pro) motif that is essential for polymerase activity, was present at position 363–365 in the WYSV L protein. The other five conserved motifs (Premotif A, motifs A–D) were also similar to those of the rhabdorhaviruses, indicating their fundamental importance in the RdRp enzymatic function (Poch et al., 1989). The tested algorithms failed to identify signal peptide in the seven predicted proteins encoding by WYSV.

**Figure 6:**
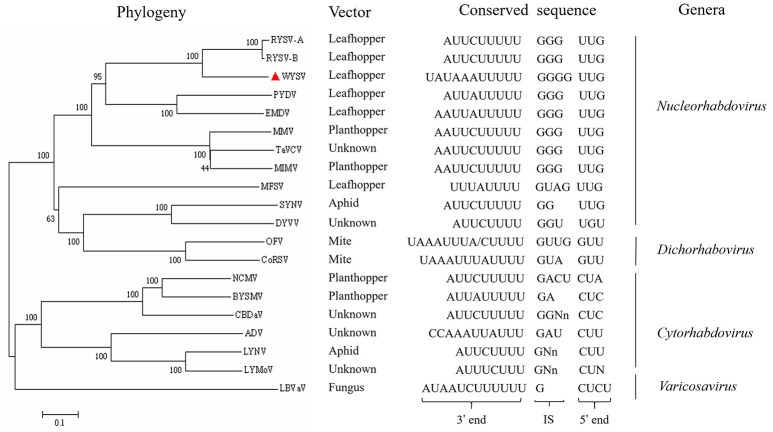
Phylogenetic relationships between WYSV and selected plant rhabdoviruses based on the amino acid sequences of the viral L protein. Sequences were aligned using Clustal W within the program MEGA 7.0 before construction of neighbor-joining trees using 1,000 bootstrap replicates. Bar indicates amino acid substitutions per site. Transmission vector and conserved intergenic sequences are listed for selected rhabdoviruses. Rhabdovirus genera, where defined, are labeled on the far right. IS, intergenic sequence. Reference virus names and the GenBank/Refseq accession numbers are as follows: rice yellow stunt virus (RYSV-A, AB011257), rice transitory yellowing virus (RYSV-B, AB516283), potato yellow dwarf virus (PYDV, GU734660), eggplant mottled dwarf virus (EMDV, KJ082087), maize mosaic virus (MMV, AY618418), taro vein chlorosis virus (TaVCV, AY674964), maize Iranian mosaic virus (MIMV, DQ186554), maize fine streak virus (MFSV, AY618417), sonchus yellow net virus (SYNV, L32603), datura yellow vein virus (DYVV, KM823531), orchid fleck virus (OFV, AB244418), coffee ringspot virus (CoRSV, KF812526), northern cereal mosaic virus (NCMV, AB030277), barley yellow striate mosaic virus (BYSMV, KM213865), colocasia bobone disease-associated virus (CBDaV, KT381973), alfalfa dwarf virus (ADV, KP205452), lettuce necrotic yellows virus (LNYV, AJ867584), lettuce yellow mottle virus (LYMoV, EF687738), and lettuce big-vein associated virus(LBVaV, AB075039).

Each ORF in the WYSV genome was separated by a highly conserved gene junction with the consensus 3′-UAUAAAUUUUUGGGGUUG-5′ except for the 3′/N junction (Figure 4B), which is similar to the intergenic sequences of other plant rhabdoviruses (Figure 6). The gene junction is organized into three elements: a 3′ poly-adenylation signal (element I), intergenic spacer (element II) and a transcription initiation sequence (element III), which is a common characteristic of the rhabdoviruses (Jackson et al., 2005). Element I consisted of a poly-U track of five residues in all gene locations, except that of the 3′ leader/N and P6/L junction, which had three and two residues, respectively. Element II consisted of a GGGG residues except for the 3′ leader/N and P/P3 junctions, and no G residue was found in the P6/L junctions. Finally, element III, likely the transcriptional start site, began with UUG in all cases except for L/5′ trailer junctions, which began with UUA. The complete sequences of the N/P, P3/M and M/G junctions were identified and found to be the same (Figure 4B). Compared to the conserved intergenic regions of other plant rhabdoviruses, sequences of both element II and III were most similar to those of nucleorhabdoviruses (Figure 6).

### Phylogenetic analysis

A total of 20 plant-infecting rhabdovirus L protein aa sequences (WYSV and 19 representative members of the genera) were used to infer the phylogenetic relationships. The result reveals that WYSV is most closely related to two strains of RYSV, which is consistent with the aa sequence identity obtained values (Figure 6). WYSV, RYSV-A and RYSV-B were located in one subcluster in the nucleorhabdoviruses in a clade that also contains EMDV and PYDV. These five viruses are leafhopper transmitted, while the planthopper-transmitted viruses MMV, MIMV, and TaVCV, which its vector is unknown, were located in a different subclade. Similar phylogenetic relationships were inferred when NJ trees were constructed using either the N or P aa sequences (data not shown). The analyses highlight the close evolutionary relationships between WYSV and RYSV, which are also reflected in the similarities in genome organization between the two viruses.

## Discussion

Several viruses in the family Rhabdoviridae are important pathogens of cultivated plant species in the family Gramineae. In this study, we characterized the complete genome sequence of a novel wheat-infecting nucleorhabdovirus which is 14,486 nt in length. Phylogenetic relationships revealed that this emerging virus represents an evolutionarily distinct lineage in the genus *Nucleorhabdovirus*. During 1950s−1960s, a putative nucleorhabdovirus wheat striate mosaic virus, transmitted by another leafhopper (*Endria iniminca, Delphacodes pellucida*), was reported in Canada and Europe (Lee, 1963; Lee and Bell, 1963), but no molecular data are available until now. To our knowledge, this is the first evidence that a leafhopper-transmitted nucleorhabdovirus can naturally infect wheat which cause yellow striate symptoms. Meanwhile, our biological experiments also determined that it can infect barley and oat.

Although rhabdoviruses are generally known to share common morphological features and a canonical gene organization encoding five structural proteins in the order 3′-N-P-M-G-L-5′, the genomes of plant-infecting rhabdoviruses may also have more complex organizations and contain additional ORFs that encode putative accessory proteins at different positions in their genomes (Walker et al., 2015). So far, all sequenced plant viruses in the genera *Nucleorhabdovirus* and *Cytorhabdovirus* have one to four additional genes between the P and M genes, such as the sc4 gene of SYNV, 4b gene of LNYV, p3 gene of RYSV and LYNV (Scholthof et al., 1994; Huang et al., 2003; Dietzgen et al., 2006). Additionally, some rhabdoviruses also contain an additional transcriptional unit encoding a small protein between the G and L genes, such as the p6 gene of RYSV and ADV (Huang et al., 2003; Bejerman et al., 2015). Our analysis reveals that WYSV carries seven non-overlapping genes on the negative-sense viral genome. In addition to the typical rhabdovirus genes N-P-M-G-L, there is an additional reading frame between the P and M genes that encodes a protein of 37.7 kDa and another between the G and L genes that encodes a protein of 13.1 kDa, indicating that the genome of WYSV encodes seven proteins arranged in the order 3′-N-P-P3-M-G-P6-L-5′. The same organization has been observed in other rhabdoviral genomes, such as RYSV (Huang et al., 2003), which is a monocot-infecting nucleorhabdovirus transmitted by leafhoppers, and ADV, which is a dicot-infecting cytorhabdovirus vectored by unknown vector (Bejerman et al., 2015), as well as the recently characterized rice stripe mosaic virus (RSMV), which is a monocot-infecting cytorhabdovirus vectored by leafhoppers (Yang et al., 2016). The first additional reading frame WYSV P3, shares high aa sequence identity (52.2%) with P3 of both RYSV-A and RYSV-B, which acts as a movement protein (MP) (Huang et al., 2005; Hiraguri et al., 2012). The second accessary gene product of WYSV, P6, shares the highest identity with that one encode by RYSV (29.5% identity). The P6 of RYSV is 63 nt shorter than WYSV P6 and is a systemic RNA silencing suppressor, which prevents RNA silencing amplification by interacting with RDR6 (Guo et al., 2013). The function of WYSV P6 is currently unknown. In summary, the six translated sequences of WYSV N, P, P3, M, G and L ORFs showed significant homology to the corresponding ORFs of RYSV except for the P6 ORF, which was far more diverse. Meanwhile, phylogenetic analysis also supports that WYSV and RYSV may have a common ancestor. Furthermore, based on the related prediction algorithms, some NLSs and NESs were successively identified in the ORFs encoded by WYSV. This suggested that WYSV viral replication and morphogenesis likely occur within the nucleus of infected cells, which were similar to that of the other nucleorhabdoviruses (Tsai et al., 2005; Goodin et al., 2007; Dietzgen et al., 2015).

In addition, the sequence of the conserved intergenic junction, which is common to all WYSV genes, has the highest similarity with nucleorhabdoviruses. Each ORF is separated by a noncoding region of 157–372 nt that contains a conserved nucleotide sequence identified as UAUAAAUUUUUGGGGUUG. The only exception to these consensus motifs is the UUA transcription initiation sequence that regulates L mRNA expression. The leader and trailer sequences are 76 and 258 nt, respectively. In comparison with the leader or trailer sequences of other rhabdoviruses, the leader of WYSV is the shortest, and there is no obvious sequence homology between the corresponding regions except for a few terminal sequences. As is typical for all rhabdoviruses, the 3′ and 5′ end sequences of WYSV are complementary and can form a putative panhandle structure thought to be involved in genome replication (Jackson et al., 2005).

Most plant rhabdoviruses are dependent on transmission by hemipteran insects, so their prevalence and distribution is closely linked to the ecology and host preferences of their vector and virus–vector interactions are highly specific (Jackson et al., 2005; Mann and Dietzgen, 2014). WYSV is transmitted specifically by the leafhopper *P. alienus* and other vectors tested where not able to transmit the virus which supports the virus-vector specificity regarding the rhabdovirus transmission. Like WYSV, other rhabdoviruses such as, maize fine streak virus (MFSV) (vector: *Graminella nigrifrons*) (Redinbaugh et al., 2002), RYSV (vector: *Nephotettix cincticeps*) (Hiraguri et al., 2010), RSMV (vector: *Recilia dorsalis*) (Yang et al., 2016) and WASMV (vector: *Endria inimical* and *Elymana virescens*) (Sinha and Chiykowski, 1967; Seifers et al., 1995), are also cereal-infecting rhabdoviruses transmitted by different species of leafhoppers.

*P. alienus* is a major agricultural pest, which annually causes significant yield losses of wheat in Asia, Europe and North America (Derlink et al., 2018). It vectors the cereal-infecting WDV, which belongs to the genus *Mastrevirus* within the family Geminiviridae, and causes huge economic losses to wheat and barley production across Europe, Africa and Asia (Köklü et al., 2007; Kundu et al., 2009; Kumar et al., 2014; Liu et al., 2014). WDV was first isolated in wheat fields in Shanxi Province in China in 2007 and has become widely distributed in northern China in recent years, especially in northwestern China (Xie et al., 2007; Liu et al., 2012; Zhang et al., 2017b). The novel wheat nucleorhabdovirus WYSV in this report and geminivirus WDV are both transmitted by *P. alienus* in a persistent manner, but they differ in that WDV is nonpropagative in the insect. Among the *P. alienus* leafhoppers and wheat plants that we collected in the WYSV-infected field, a few were co-infected by both viruses (data not shown). To date, no studies have been conducted to elucidate the consequences of any interactions between a geminivirus and plant-infecting rhabdovirus in their natural vector insect. Further research is necessary to explore the possible interactions between these two diverse viruses of cereal crops within their natural vector, specifically when the two viruses are simultaneously acquired.

Taken together, our results highlight a novel nucleorhabdovirus that infects cereal crops via a leafhopper and may thus be an emerging threat to cereal production. Although the genomic sequences of WYSV are known, more biological and ecological features remain to be elucidated before it causes a devastating epidemic. A rapid detection method for WYSV in wheat plants and vector leafhoppers is thus urgently needed, but methods to control the virus depend on further characterization of the virus with regards to its translation and replication strategies, transmission mechanism, pathogenesis and host responses. A newly developed reverse genetic system suitable for plant rhabdoviruses will enable the study of their functional properties *in vivo* in the plant and insect hosts (Jackson and Li, 2016).

## Author contributions

XW: Conceived and designed experiments; YL and XW: Collected samples; YL, ZD, HW, SZ, and MC: Performed the experiments and analyzed data; YL and MC: Discussed the results and drafted the manuscript; XW and MC: Revised the manuscript. All authors read and approved the final manuscript.

### Conflict of interest statement

The authors declare that the research was conducted in the absence of any commercial or financial relationships that could be construed as a potential conflict of interest.
