# Supplementary material for: Identification and Characterization of Wheat Yellow Striate Virus, a Novel Leafhopper-Transmitted Nucleorhabdovirus Infecting Wheat
Source: Front Microbiol. 2018 Mar 14;9:468. doi: 10.3389/fmicb.2018.00468 (PMC5861215; doi:10.3389/fmicb.2018.00468)
Supplement: Supplementary file 1 [file Presentation1.PDF]

Supplementary material

Identification and Characterization of Wheat Yellow Striate Virus, a Novel Leafhopper-transmitted *Nucleorhabdovirus* Infecting Wheat in China

Yan Liu[1], Zhenzhen Du[1], Hui Wang[1], Song Zhang[2], Mengji Cao[2*], Xifeng Wang[1*]

[1] State Key Laboratory for Biology of Plant Diseases and Insect Pests, Institute of Plant Protection, Chinese Academy of Agricultural Sciences, Beijing 100193, China

[2] National Citrus Engineering Research Center, Citrus Research Institute, Southwest University, Chongqing 400712, China

*Corresponding author:

Xifeng Wang, E-mail: xfwang@ippcaas.cn;

Mengji Cao, E-mail: caomengji@cric.cn

TABLE S1 | List of primers used in this study

No.	Primer	Sequence (5' to 3')	Base (nt)	Size (bp)
1	1F-P121	CAAGCGTCGTCACATTCCTA	20	1611
	1R-P1731	ATTGTGCTCTAGTTCTGGCGTTA	23	
2	2F-P1671	ACCAAAGGGCAAGAGATGCTG	21	1797
	2R-P3467	GCTGGTTGTCTTTCTGTCTTTCG	23	
3	3F-P3321	TATTGACTAAAGGGGAGGTTGTTACA	26	1533
	3R-P4853	TTCATCATCTGTGTGGGTCATCC	23	
4	4F-P4831	GGATGACCCACACAGATGATGAA	23	1558
	4R-P6388	AGGCTGTAGAACCATGTATTGCA	23	
5	5F-P6282	ATTGAAGCTGATGATAGACTCGGTT	25	1505
	5R-P7786	CTTGCTTGTTGTCTAGGCTCTTGTC	25	
6	6F-P7647	CTGAGCCGGATAAGGGATACAT	22	1634
	6R-P9280	CGAGGTGTAGGAGATAATGCTTCA	24	
7	7F-P9183	ATGGGGAATGAAGGATACAATG	22	1592
	7R-P10774	TGTAAGGTCATAGAGGTGTTGTTCG	25	
8	8F-10750	CGAACAACACCTCTATGACCTTACA	25	1532
	8R-P12281	GCAGGATTCATGAGCGTGGA	20	
9	9F-P12222	GTAATGCTCCTTATGAACAACGG	23	1521
	9R-P13742	CACCTGACGGGACTGCTTGT	20	
10	10F-P13715	TATGAGTTACAAGCAGTCCCGTC	23	745
	10R-P14459	GTTTCTTGTATGGATATGGGGTCAT	25	
11	11F-P1	ACACCACCAGACAACAACTGCA	22	1732
	1R-P1731	ATTGTGCTCTAGTTCTGGCGTTA	23	
12	10F-P13715	TATGAGTTACAAGCAGTCCCGTC	23	772
	11R-P14486	ACACCACCATATCCAAAACCG	21	
13	5R-GSP1	CATTAGCATAGTTAGCAA	18	
	5R-GSP2	GGTGCCAGTGAAGTTGTTGTTG	22	
	5R-GSP3	TTAGGAATGTGACGACGCTTGC	22	
14	3R2-GSP2	CCCAACCAACCACAACTGCC	20	
	3R2-GSP3	TGTTGGGTCTTGGGATAATGA	21	
	AUAP	GGCCACGCGTCGACTAGTAC	20	
